# A Bayesian Framework to Account for Misclassification Error and Uncertainty in the Estimation of Abortion Prevalence

**DOI:** 10.1111/sifp.70053

**Published:** 2026-05-05

**Authors:** Marija Pejchinovska, Monica Alexander

## Abstract

Obtaining reliable estimates of the prevalence of induced abortion remains a significant challenge in abortion research. Recently, one indirect, survey‐based technique for measuring abortion outcomes, the confidante method, has gained particular attention. The method has been applied in various social and legal contexts; however, its efficacy has not been uniformly established. Increasingly, focus has shifted to assessing the method's key assumptions and quantifying the biases that arise from violations of them. We propose a general statistical framework to conceptualize and quantify the impact of biases on measuring abortion prevalence from such surveys. Specifically, we define the relationship between observed and true abortion prevalence based on misclassification error related to the sensitivity and specificity of the survey instrument. This formulation leads naturally to a Bayesian modeling approach to estimate abortion prevalence, allowing for differing knowledge of and different levels of uncertainty about the misclassification parameters to be incorporated in the modeling process, with that uncertainty being propagated through to the final estimates. We illustrate our framework and modeling approach on data from an application of the confidante method in Uganda in 2018, where we account for systematic differences in confidante abortion reports based on the self‐reported abortion experiences of survey respondents.

## INTRODUCTION

Effective monitoring of abortion outcomes is highly relevant for various public health considerations, including programs, policies, and interventions aimed at improvements in access to safe reproductive health services and the allocation of necessary resources. Obtaining reliable estimates of these indicators, however, has proven quite challenging and persists as one of the most fundamental problems in abortion research.

To date, a range of methods and data collection strategies have been employed in the estimation of the incidence of induced abortion. These approaches fall into two broad categories, direct and indirect methods. Direct methods generally include querying official statistics, using data from health facility surveys, or directly asking women about their abortion experiences in population‐based surveys. These approaches, however, are not feasible in all countries, and data from official statistics and health facilities surveys can often be incomplete or inaccurate in many low‐income, low‐resource settings (see Lara et al. 2004; Mensch, Hewett, and Erulkar 2003). While such measurement issues are not unique to abortion, they are often exacerbated by the legal and cultural contexts of abortion. Moreover, self‐reports of induced abortions, obtained by directly asking women, have been found to significantly underreport the true prevalence of the procedure (see Rossier 2003 for a detailed discussion). Despite researchers' many efforts to encourage full disclosure and refine survey instruments to preserve anonymity, underreporting has remained prevalent.

To address some of the limitations of direct methods, various types of indirect methods have been proposed. These include variations of the item counting technique, such as the list experiment and double list experiment (e.g., see Moseson et al. 2021; Sedgh and Keogh 2019; Keogh et al. 2020; Bell and Bishai 2019); the randomized response technique and its variants (Lara et al. 2004, 2006; Tezcan and Omran 1981; for challenges and limitations see Coutts and Jann 2011); the abortion incidence complication methodology (AICM) (Singh, Prada, and Juarez 2010; for applications see Singh et al. 2019; Giorgio et al. 2020; Polis et al. 2020; Keogh et al. 2020; for the modified AICM, see Sedgh and Keogh 2019); and methods that rely on social network‐based samples, most notable among which are the anonymous third‐party reporting method (Rossier 2010; originally developed in Rossier 2002), the best friend approach (Yeatman and Trinitapoli 2011), the confidante method (Sedgh and Keogh 2019; initial version discussed in Rossier et al. 2006), and the network scale‐up method (Sully, Giorgio, and Anjur‐Dietrich 2020).

In recent years, methods relying on social network‐based samples—often referred to as third‐party reporting (TPR) of close ties, which capture respondents’ reports on the abortion experiences of close confidantes—have gained significant attention. The underlying philosophy of TPR methods is that women are more likely to honestly report the experiences of their close friends, rather than their own, on topics as sensitive as abortion, particularly in settings where access to abortion is considerably limited or the procedure is illegal or highly socially stigmatized.

In this work, we focus our attention in particular on the estimation of abortion prevalence using the confidante method, a TPR method where information on abortion experiences is collected on up to three close confidantes of the survey respondent. The approach has been found to outperform direct reporting; however, its efficacy has not been uniformly established. A challenge with the confidante method, and indeed all TPR methods, is that resulting estimates can often be subject to several types of biases. Aspects of the survey questionnaires and data gathering approaches could impact the quantities in the numerator and denominator of a standard abortion prevalence or incidence, potentially giving rise to biases such as transmission bias, selection bias, and barrier bias. This has revealed a real need in the field to carefully assess the method's underlying assumptions and any biases that may result from violations in those assumptions. Since the development of the confidante method, there have been a handful of studies that discuss, examine, and, where feasible, adjust for the sources of bias associated with the confidante method specifically, and TPR methods more broadly (see, for instance, Keogh et al. 2020; Stillman et al. 2020; Bell et al. 2020; Giorgio, Sully, and Chiu 2021; Owolabi et al. 2023).

Motivated by this, we introduce a statistical framework which helps us reframe some of the resulting biases in the context of misclassification error. We draw inspiration from existing work in the medical sciences and epidemiology and define a framework which allows us to tie the estimation of abortion prevalence from a confidante survey to such survey instrument attributes as *sensitivity* and *specificity*. Sensitivity and specificity are often seen as important measures of the quality of a binary classification scheme, and there is large, decades‐spanning literature on the need to account for such measures in the context of faulty instruments, diagnostic tests, and survey questions, even when no gold standards for comparison exist (for some early examples, see Buck and Gart 1966; Staquet et al. 1981; Valenstein 1990; for more recent works see Georgiadis et al. 2003; Högg et al. 2017; Johnson, Jones, and Gardner 2019). And while such conceptualization allows us to account for misclassification in the survey instrument, our knowledge of the sensitivity and specificity of the instrument is likely to remain uncertain. We demonstrate how Bayesian estimation enables us to address uncertainty around given parameters through the use of prior distributions, which help to encode knowledge about misclassification.

An important aspect of our approach is recasting violations in the existing assumptions of the confidante method using notions of sensitivity and specificity. While we do not set out to address and make adjustments for all potential biases identified in the confidante method, and in particular biases associated with nonrepresentativeness of the confidante sample (see the Key Assumptions of the Confidante Method section for biases associated with the denominator), our framework offers a more principled way of estimating abortion outcomes by incorporating measured demographic characteristics, integrating varying levels of information about the survey's sensitivity and specificity as well as misclassifications in the outcome (including differing patterns of misclassification), and more systematically accounting for sources of uncertainty.

We illustrate our framework and modeling approach on data from an application of the confidante method in Uganda in 2018, carried out through the Performance Monitoring for Action (PMA) project. Our focus is on the estimation of abortion prevalence; however, the modeling framework could also be used for the estimation of annual abortion incidence (which we illustrate separately in Appendix E of the Supporting Information). We exploit particularities in the Uganda abortion data to highlight the potential benefits of the framework and the Bayesian model. While we demonstrate this framework using data collected through the confidante method, it is broadly applicable to other TPR methods or any survey data with a binary outcome measuring a sensitive behavior.

The remainder of the paper is organized as follows: The second section provides background on abortion incidence estimation using the confidante method, lays out the method's key assumptions, and highlights some of the existing work in assessing and adjusting for violations of those assumptions. In the third section, we describe a statistical framework for understanding measurements from a confidante method survey through the lens of sensitivity, specificity, and misclassification of the survey instrument. We integrate this framework within a Bayesian modeling approach, which we describe in the fourth section through an application of the confidante method in Uganda. Finally, the Conclusion and Discussion section presents some limitations and possible extensions of our approach.

## BACKGROUND ON ABORTION INCIDENCE ESTIMATION USING THE CONFIDANTE METHOD

The confidante method focuses on a small, fixed number (usually up to three) of respondents' closest confidantes and their abortion experiences. The method stipulates that the relationship between the respondent and her confidantes is one of reciprocity; in other words, confidantes are defined to be the women who share their secrets with the respondent and with whom the respondent shares her secrets (Sedgh and Keogh 2019).

Since 2018, the confidante method has been applied in various legal and social contexts in countries in South and Southeast Asia and parts of Africa. Applications include a number of surveys facilitated by the Performance Monitoring for Action 2020 (PMA2020) initiative and fielded in 2018 in Nigeria, Cote D'Ivoire, and the state of Rajashtan in India (Bell et al. 2020), in Ghana (Keogh et al. 2020), and in Uganda and Ethiopia (Giorgio, Sully, and Chiu 2021), along with a separate study carried out in Java, Indonesia (Stillman et al. 2020). The methodology has been found to be a considerable improvement over direct reports in all applications. However, establishing the efficacy of the method and the reliability of its estimates has proven difficult. Resulting estimates have often been found to be context‐dependent, with the method's performance affected by the legal and social context of abortion in each setting.

Despite the mixed performance of the method, it is still largely considered a useful estimation approach. The current consensus, however, seems to be that more evidence from applications of the method in varied cultural and legal contexts is needed to establish its efficacy and usefulness in measuring abortion (see Giorgio, Sully, and Chiu 2021; Bell et al. 2020; Keogh et al. 2020; Stillman et al. 2020). An important existing hurdle in assessing the method's efficacy is that despite its many applications, the specifics of each application have varied across studies, as have the analytic approaches used in the estimation process (Owolabi et al. 2023), making comparisons harder.

### Key Assumptions of the Confidante Method

Given that violations in the assumptions of the confidante method can have potentially significant effects on the final estimates, there has been an increased effort recently towards providing a better understanding of key assumptions. Among these most recent works, Giorgio, Sully, and Chiu (2021) offer the most comprehensive discussion of the assumptions underlying the method. And more recently, Owolabi et al. (2023) have relied on these same key sociological assumptions in their comparative analysis of existing approaches to bias assessment and adjustment. Because a discussion of these is relevant for the general framework we propose in the sections that follow, we use this section to briefly highlight the six key assumptions of the confidante method.

In order to be consistent with most recent efforts around the confidante method, in Table 1, we present the assumptions exactly as they appear in Giorgio, Sully, and Chiu (2021) (for a more detailed discussion, see the original paper). Note that assumptions N1–N3 affect the measurement of the numerator of abortion incidence, while D1–D3 refer to assumptions generally concerning the denominator.^1^

**TABLE 1:** Main assumptions of the confidante method

Assumption number	Assumption
Numerator	
N1	Respondents and confidantes share information about their abortion experiences
N2	Respondents have complete knowledge of confidantes' abortions ‐ implies no *transmission bias*
N3	Respondents have the willingness and ability to share knowledge of confidantes' abortions ‐ implies no *social desirability bias* or *recall bias*
Denominator	
D1	Respondents select confidantes with homophily—violations of this assumption would result in *selection bias*
D2	No systematic differences between respondents who report zero and those who report at least one confidante—violations here could potentially lead to biased estimates as a result of *barrier effects*
D3	Confidantes' inclusion in the surrogate sample is independent of their abortion status—assumes no *popularity bias*

### Existing Work in Assessing and Adjusting for Violations to Key Assumptions of the Method

Among the studies examining the confidante method (Giorgio, Sully, and Chiu 2021; Keogh et al. 2020; Owolabi et al. 2023; Stillman et al. 2020; Bell et al. 2020), approaches to assessing violations in the method's assumptions and adjusting for the resulting biases have largely been unsystematic (for a more detailed discussion and comparative analysis, see Owolabi et al. 2023). Here, we highlight some of the more relevant assumption violations and corresponding adjustments that have been documented in the literature.

When thinking of the numerator, the assumption that respondents have complete knowledge of their confidantes' abortion experience (Assumption N2) has been particularly consequential. All studies referenced in this section document violations of this assumption and conclude that women might not fully observe their confidantes' abortion experiences. Adjustments here have varied, however, from model‐based imputation of the abortion experiences of the “missing” confidantes as partial remedy for transmission bias (Bell et al. 2020), to directly incorporating a version of an inflation factor—a proxy for visibility of abortion—calculated using the definition of reciprocity in the respondent–confidante dyad and information on proportion of respondents' abortions that are visible to confidantes (Giorgio, Sully, and Chiu 2021; Keogh et al. 2020; Stillman et al. 2020).

Many of the applications of the confidante method have revealed violations of the assumption of homophily (Assumption D1) as well as systematic differences between the respondents that report zero confidantes and those that report at least one, a violation of assumption D2 (Giorgio, Sully, and Chiu 2021; Keogh et al. 2020; Stillman et al. 2020; Bell et al. 2020). Both of these issues can lead to a nonrepresentative surrogate sample. In the cases where adjustments to violations of assumption D2 have been made, they have generally involved some form of “adding” the missing confidantes and their abortion experience, often in a model‐based way, to the confidante sample (Keogh et al. 2020; Bell et al. 2020). We find such adjustments particularly tricky as it is possible that they introduce biases of unknown direction and magnitude, which would be difficult to evaluate. To our knowledge, no study has assessed or adjusted for popularity bias, a violation of assumption D3.

## LINKING TRUE AND OBSERVED ABORTION PREVALENCE THROUGH A MISCLASSIFICATION FRAMEWORK

Our aim here is to link the key assumptions of the confidante method to a statistical framework that describes the observed abortion prevalence in terms of misclassification errors. In this section, we highlight only the main points and introduce basic notation, while relevant derivations and additional details are described in Appendix A (part of the Supporting Information).

The outcome of interest is a binary variable indicating whether or not a woman has ever had an abortion. Here, we focus on women's lifelong abortion experiences. The specific choice of measured response, however, does not have a material impact on how we define concepts—they are broadly applicable to any binary outcome—nor does it pose any issue for the model we propose in subsequent sections. Given, however, that annual abortion incidence is often of interest, Appendix E of the Supporting Information provides the results of the application of our modeling framework to the estimation of annual incidence.

We make a distinction between an underlying true, but possibly unobserved, abortion experience for every individual i, which we denote as $Y_{i}^{\;\mathrm{TRUE}}$, and an observed abortion status measured through a potentially imperfect survey instrument, denoted as $Y_{i}^{\;\mathrm{OBS}}$, each one of which can take on the value 0 or 1, where 1 would indicate individual i has had or is reported as having had an abortion.

Ideally, we would like to observe the true underlying probability of an abortion, which we denote as $\gamma_{i}^{\mathrm{TRUE}}$. In our imperfect reality, however, we only observe the probability of reporting an abortion, denoted as $\gamma_{i}^{\mathrm{OBS}}$.

Because survey questionnaires—either ones directly eliciting self‐reports from respondents or those seeking information on their close ties—are imperfect survey instruments, they are likely to measure the outcome of interest with some error. In the case of a binary (yes/no) response, such errors are typically referred to as misclassifications (Gustafson 2003; Yi 2017). Because of the sensitive nature of abortion, such misclassifications likely lead to serious underreporting and consequently a general underestimation of the abortion prevalence. This is particularly true of direct reports but can also be true of reports on close ties.

In this work, we focus on recasting some of the biases that arise from violations in the method, particularly those concerning the numerator. We propose examining the misclassification of the confidante survey instrument through the notions of sensitivity and specificity, where we define the sensitivity (SE) of the instrument as the probability of observing a true underlying abortion, and the specificity (SP) as the probability that the instrument correctly captures a true underlying absence of abortion.

Thinking about misclassification in the survey responses in terms of SE and SP of the survey instrument allows us to relate the true abortion probability—the quantity we are truly interested in—with what we observe in the survey. Namely,

(1) $$\gamma_{i}^{\mathrm{TRUE}}=\frac{\gamma_{i}^{\mathrm{OBS}}-\left(1-\text{SP}\right)}{\text{SE}-(1-\text{SP})}.$$

Note that in Equation (1), SE and SP are taken to be constant, meaning that we are effectively assuming that the probability for error or misclassification does not depend on any other factors apart from the true underlying abortion status. In the literature, this is known as *non‐differential misclassification* (Yi 2017). However, in the abortion context, the assumption of constant misclassification is likely to be too strong. In contrast to non‐differential misclassification, *differential misclassification* occurs when the probability of misclassified or erroneous response is different for different subpopulations. For instance, based on individual characteristics (e.g., age, educational level, marital status), different women in the survey may have a differential propensity to disclose their true abortion status (or that of their confidantes). This matters, as failing to account for differential misclassification could make it difficult to learn the true distribution of abortion across various characteristics, leading us to potentially over‐ or underestimate the abortion behavior of specific subpopulations. Allowing for SE and SP to vary by levels of a specific covariate or a combination of covariates involves a simple extension of Equation (1), details of which can be found in Appendix A.1 of the Supporting Information. One challenge with the expression in (1), including its stratified version, is that it relies on knowing the true values of SE and SP. In practice, we do not usually have a way of truly measuring the SE and SP of a survey questionnaire, in which case we are faced with the challenge of using estimates of these quantities. An additional difficulty is that there is no clear way to account for the uncertainties associated with any estimates of SE and SP we might be interested in using. In the Model section, we illustrate how a Bayesian framework goes some way towards remedying such issues by modeling uncertainty in the misclassification parameters.

### Relating Sensitivity and Specificity to the Method's Key Assumptions

The sensitivity and specificity of the confidante survey instrument largely relate to the method's assumptions concerning the numerator (N1–N3), though they can, to some extent, have an impact on the assumptions about the denominator (D1–D3) as well. We note that in the modeling framework that we propose, biases stemming from violations of the assumptions regarding the denominator are not directly addressed within the SE/SP mechanism, but are rather, where feasible, considered in a post‐estimation step, specifically through techniques such as poststratification.

In general, in settings where abortion is legally restricted or highly socially stigmatized, most attempts to measure abortion result in some level of underreporting, which, in turn, translates into downward‐biased estimates. This helps explain some of the dynamics in the sensitivity and specificity of the survey instrument. Because of the considerable social stigma frequently associated with abortion, women are unlikely to falsely report having had an abortion. As a result, we expect the specificity of the survey instrument, meaning the instrument's ability to correctly identify those women who have never had an abortion, to be quite high. In particular, this should be true in the case of self‐reports. However, this may not always be true in the case of the sample of surrogates due to possibly imperfect knowledge among respondents of their friends' abortion experiences. On the other hand, it is reasonable to expect that the survey instrument would have poorer sensitivity. Social desirability biases drive women to be less likely to truthfully disclose abortion experiences, especially their own. The higher false negative rate that could result from this would then pose challenges to valid estimation.

To examine SE and SP in an even more granular way, and more specifically in the context of the surrogate sample, we refer back to the six key assumptions of the confidante method. Table 2 presents a collection of hypothetical situations which lead to violations of assumptions N1–N3 and describes the resulting effects on SE and SP.

**TABLE 2:** A set of hypothetical scenarios illustrating the effects of particular violations in assumptions N1–N3 on the SE and SP of the survey instrument

Scenario Number	Assumption	Scenario Setup	Effect on SE	Effect on SP
1	N1 ‐ Sharing of information on abortion	Abortion is illegal and access to abortion services is largely restricted. Women reach out to trusted social ties to learn of ways to access abortion. Abortion is stigmatized but accepted among members of women's social networks, and experiences are shared directly among close social ties.	Likely increase in SE	SP remains high
2	N2 ‐ Complete knowledge of confidantes' abortions	Access to abortion is limited, but women might be able to find service providers without reaching out to members in their social network. The procedure is, however, highly socially stigmatized, and most women who have abortions share their experience with only a handful of their trusted friends, if at all.	Likely decrease in SE	SP remains high
3	N2 ‐ Complete knowledge of confidantes' abortions	Abortion is legally restricted and highly stigmatized, but information on abortion can still be accessed. Certain women can access an abortion without the help of their networks, either by reaching out to various community programs or because information on abortion services are publicly available. Direct sharing among friends is more limited and women can often learn of their friends' abortion experiences indirectly, from other members in their social network.	Likely decrease in SE	Potential decrease in SP
4	N3 ‐ Willingness or ability to disclose confidantes abortions	Abortion is illegal and highly socially stigmatized. Women may need to reach out to their social networks for information on access, but most women are very reluctant to disclose the abortion experiences of their friends even if they have complete knowledge of it.	Likely decrease in SE	SP remains high

Broadly speaking, in much of the literature on TPR of abortion (see Rossier et al. 2021), there seems to be a suggestion that assumption N1 (sharing of information about abortions among social ties) is largely satisfied, particularly in low‐ and middle‐income countries, in setting where abortion laws are stricter or where access to abortion in the formal or informal health sectors is severely restricted. The degree of the impact any violations in N1 might have on SE and SP will largely depend on the degree of sharing of information. For instance, consider Scenario 1 in Table 2. When women are forced to rely on their social networks to access abortion, there is generally a higher degree of sharing of information on abortion within networks. This increased sharing of information is likely to push SE upward. It is worth noting, however, that the degree of sharing would be difficult to quantify in practice. Furthermore, violations in assumption N1 may occur concomitantly with violations in assumptions N2 or N3, making their impact difficult to distinguish.

Violations in assumption N2 (presence of transmission bias or imperfect knowledge) can have a significant impact on the SE of the instrument. In particular, transmission bias tends to drive down the SE of an instrument by increasing the number of false negatives. This bias will, in part, be driven by the visibility of abortion in the specific setting where the study is carried out. For example, Scenario 2 in Table 2 depicts a setting in which access to abortion is possible without reaching out to one's network and where only some women share their experience with friends. In this particular setup, the SE is likely to be lower. Occasionally, transmission bias may occur in situations where respondents learn of their confidantes' abortion experiences indirectly, meaning through other members in their social network—this is Scenario 3 in the table. Such bias resulting from potentially imperfect or flawed knowledge can impact SE, but also SP of the instrument, and is likely to lead to a decrease in both quantities. To the extent that a particular confidante method survey offers a way of quantifying the degree to which indirect sharing of information about abortions occurs within social networks, such information could be incorporated in both the SE and SP of the instrument. It should be noted, however, that this type of transmission bias, resulting from indirect sharing, is somewhat rare. Nearly all abortion knowledge occurs through direct sharing.

Recall bias (a violation of assumption N3) is slightly less impactful when the outcome of interest is the lifelong abortion experience. Nevertheless, respondents may be unaware or have difficulty recalling friends' abortions that have happened a long time ago. This will have an effect on the SE of the instrument, likely biasing the estimates downward.

While assumptions affecting the denominator, D1–D3, can have a serious impact on the estimates, their effects on the SE and SP of the survey instrument are a bit more complex. In general, assumptions D1–D3 are largely concerned with the representativeness of the confidante sample, and some violations of these assumptions (for instance, some forms of selection bias) can be addressed, at least partly, through re‐weighting of the sample using carefully constructed weights, such as poststratification weights. This is only a partial solution, however, as we can only make such adjustments on the basis of measured covariates, making this approach not very effective in dealing with barrier effects or popularity biases. There are mechanisms that can affect the denominator, and, in an indirect way, the SE or SP of the instrument, which might not fall neatly under a specific denominator assumption violation. For instance, a situation where the interviewer inadvertently primes the respondent to think of women who have had an abortion prior to fielding the survey instrument (leading to a violation in assumption D3), may as a result impact respondent's willingness to disclose the abortion experience of their confidantes (violation in N3), which would in turn impact the instrument's SE. Similarly, how respondents understand the definition of “confidante” stipulated in a given study questionnaire may impact which close ties they report on in the surrogate sample, adding to the issues of nonrepresentativeness of that sample. If this is also in some way associated with respondents' propensity to disclose their abortion experience or that of their confidantes, this can impact the SE and possibly SP of the instrument. Additionally, it is possible that the number of potential confidantes identified by those more socially connected respondents, and consequently the confidantes they chose to report on, may also impact the representativeness of the surrogate sample. If, for instance, having a larger social network of women of reproductive age who satisfy the definition of confidante is associated with a higher propensity to report abortion experiences, this in turn will increase the SE and SP of the instrument.

## Application to Data from Uganda

We implemented our framework in a particular application of the confidante method in Uganda in 2018. For context, in Uganda, abortion is illegal, except in cases where the mother's life is in danger. Despite recent efforts to expand the list of legal exceptions, the inconsistency in how the abortion law is interpreted and enforced, coupled with high societal stigma surrounding abortions, has nevertheless significantly hampered Ugandan women's ability to access safe abortion (Guttmacher Institute 2017).

### Data

The data used in this analysis were collected through a survey conducted by the Performance Monitoring for Action (PMA2020) project (formerly Performance Monitoring and Accountability project) as part of a broader family planning initiative (Zimmerman et al. 2017). We use the 2018 Round 6 of the PMA survey in Uganda (Makerere University and Bill & Melinda Gates Institute 2024).^2^ The survey uses a two‐stage cluster sample design with urban–rural as a strata, relying on enumeration areas drawn by the Uganda Bureau of Statistics, from which 44 households were randomly selected, and eligible females aged 15–49 were contacted for interviews. The survey provides a nationally representative sample of Ugandan women of reproductive age (15–49). Additional details of the sampling scheme and PMA survey methodology can be found elsewhere (PMA 2021; Zimmerman et al. 2017). The Guttmacher Institute designed a confidante method questionnaire and incorporated it in the female interviews of the 2018 survey. The confidante module was randomly administered to 2089 women and resulted in a surrogate sample of 3,268 confidantes. The data are available through Performance Monitoring for Action (PMA 2021).^3^

Previous work by Giorgio, Sully, and Chiu (2021) and subsequently Owolabi et al. (2023) has examined the application of the confidante method in Uganda. Both studies report on the annual abortion incidence rate among the respondents and the confidantes and offer an extensive discussion on quantifying potential biases in the estimates and applying adjustments. While we do not wish to reproduce the findings here, we do want to highlight some aspects and patterns in the available data that are informative for our understanding of misclassifications from the survey instrument in the Ugandan context. As part of the confidante questionnaire, researchers incorporated an item asking respondents who reported having an abortion whether they shared their abortion experience with any of their confidantes. Given the assumption that the relationship between respondents and confidantes is one of reciprocity, the item was intended to measure the broader visibility of abortion. Among the women who self‐reported abortions and also provided information on any confidantes, an estimated 70% shared their experience with at least one of their confidantes.^4^ Furthermore, when looking at the three sets of confidantes separately (i.e., the samples of first, second, and third confidantes), the proportion of respondents' abortions that was visible to each set was around 0.5. Though perhaps far from ideal, these are useful metrics for the possible size of transmission bias, and we use them to inform our understanding of the possible range of the sensitivity of the survey. We note that recent published work has attempted to estimate the visibility of abortion in the Ugandan context (Giorgio et al. 2024), placing the overall visibility at about 17%, much lower than the measure we intend to use. However, as these results pertain to visibility within a woman's entire social network and cannot easily be generalized beyond the Central region of Uganda, where the study took place, they are less applicable to our case. Below, we highlight an additional feature of the data which indicates that rates of reported abortion among confidantes differ significantly for different characteristics of the respondents, further suggesting differential sensitivity for the survey instrument.

#### Dependence between the Respondents and Confidantes Sample

**FIGURE 1:**
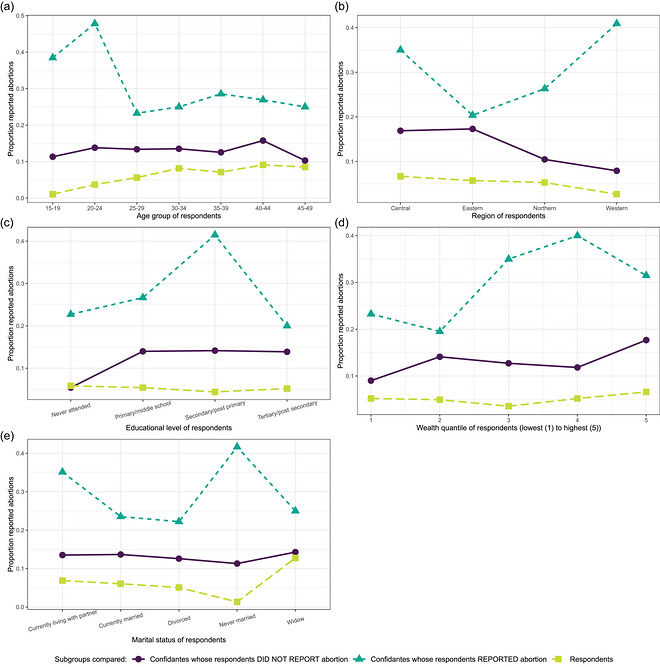
Distribution of the proportion of reported abortions among confidantes whose respondents reported having an abortion, confidantes whose respondents did not report having an abortion, and the respondents themselves

In the Uganda sample, we find that the raw (design‐unadjusted) proportion of confidante abortions varies significantly based on whether or not the confidante's respondent has herself reported having an abortion. Figure 1 shows that, as expected, the raw proportion of lifetime abortion experiences among confidantes is higher than that among respondents across a range of socioeconomic variables. However, when looking at the confidante sample only, the group of confidantes whose respondents self‐reported an abortion has a consistently higher abortion proportion compared to those confidantes whose respondents did not. This is true across a number of characteristics of the respondent—age, educational level, marital status, region of residence, and wealth quantile. Several issues might be at play here. It is possible that respondents who have had an abortion are simply more likely to know other women who have also had an abortion. This might be unsurprising, given that the method assumes respondents chose their confidantes with homophily, but it might also point to potential barrier effects, if, for instance, reporting one or more confidantes is somehow associated with a higher abortion rate. It is also possible that respondents who have had an abortion are likely to have a better knowledge of the abortion experiences of their close ties as a result of possible greater visibility of abortion among certain circles and consequently reduced levels of transmission bias. It may also be the case that women who are willing to report on their abortion are also more willing to report on the abortion of their confidantes, indicating potentially lessened effects of social desirability bias among certain groups. While it is unclear what specifically is driving this behavior, this observation points to the differential capacity of the survey instrument to capture abortion‐related information among different subgroups. A similar pattern has been documented in a study carried out in Java, Indonesia (Stillman et al. 2020). The authors there point to the fact that the abortion rate was estimated to be nearly 10‐fold higher among confidantes whose respondents reported an abortion compared to those whose respondents did not. This is a useful modeling consideration. Here, the abortion status of the respondent induces a mechanism of potential differential misclassification in the observed confidante data.

### Model

To estimate the abortion prevalence in Uganda, we develop a statistical regression model in which we model the true abortion prevalence based on the observed outcomes, a set of measured covariates, and the sensitivity and specificity of the survey instrument. Specifically, the model we use in this analysis is a logistic regression in which we embed the SE/SP framework and account for the assumed differential misclassification mechanism.

We assume the data follow a Bernoulli distribution with probability $\gamma_{i}^{\mathrm{OBS}}$. We let the model governing $\gamma_{i}^{\mathrm{TRUE}}$ be a logistic regression model, where we describe the true probability of ever having an abortion as a function of a confidante's age, educational level, and region of residence. We relate $\gamma_{i}^{\mathrm{OBS}}$ and $\gamma_{i}^{\mathrm{TRUE}}$ using a version of the expression outlined in Equation (1). The full model specification is given in Appendix A of the Supporting Information.

In this application, we impose specific conditions on the SE and SP parameters. We assume that the SP parameter remains constant across characteristics of the confidantes, as we are inclined to believe that the propensity to falsely report an abortion that did not occur is rather low and independent of measured characteristics. In contrast, informed by our preliminary analysis of the Ugandan data, we let the prior distribution for SE depend on the abortion status of each confidante's respondent. Specifically, we have $\gamma_{i}^{\mathrm{OBS}}=\gamma_{i}^{\mathrm{TRUE}}\;{\text{SE}}_{S(i)=s}+\left(1-\text{SP}\right)\left(1-\gamma_{i}^{\mathrm{TRUE}}\right)$. The indexing variable S(i) has two strata, 0 and 1. If S(i)=0, confidante i’s respondent did not report an abortion, while if S(i)=1, confidante i’s respondent reported an abortion.

In a Bayesian setting, prior distributions need to be specified for all unknown parameters. Details of this are given in equations (A10)–(A16) in Appendix A of the Supporting Information. Here, we discuss only the choices of priors assigned to SE and SP. Since both SE and SP are probabilities, with values on the interval [0,1], one possible choice for a prior distribution would be a beta distribution, Beta(η,ν) (more details on prior selection for SE and SP are provided in Appendix A.1 of the Supporting Information). Given the sensitive nature of abortion, we generally assume very few women are likely to falsely report an abortion that did not occur, and so we expect the survey instrument to have a particularly high specificity, likely very close to 1. However, we intentionally do not fix SP at 1, so as to allow for the small probability of erroneous reporting. Given that in the data we have no way of discerning whether a respondent learned about her confidantes' abortion experience directly from her confidantes or through others in her social network, we wish to be able to account for the possibility of false positives. To encode this belief in the prior distribution for SP, we assign it a Beta(200,1) prior. This implies a prior mode, or most probable prior value, of 1 and a 95% prior probability interval of (0.98, 1). This allows for a relatively high specificity of the instrument while also accommodating the possibility for imperfect knowledge or transmission bias. The choice of prior distribution for the instrument's SE was partly informed by the particularities in the Uganda data. In an ideal situation, we would like the range of possible values of the prior for SE to be guided by available information outside of the sampled data. However, this is challenging in the Ugandan context, since, to our knowledge, there is currently only limited research quantifying the visibility of abortion among various groups (Giorgio et al. 2024). The model specification describes our belief that the abortion status of each confidante's respondent induces a mechanism of differential misclassification, meaning the probability of correctly identifying a true abortion is different for those confidantes whose respondents themselves reported an abortion than for those whose respondents did not. To account for this, for those confidantes whose respondents reported having had an abortion, indicated by S(i)=1, we set a $\text{Beta}\left(542,242\right)$ prior for SE with a prior mode of 0.69 and a 95% prior probability interval of (0.66,0.72). On the other hand, for those confidantes whose respondents did not report an abortion, meaning S(i)=0 for confidante i, given that we believe the true positive rate of the instrument in such cases to be even lower, we place a $\text{Beta}\left(250,200\right)$ prior on SE, implying a prior mode of around 0.56 and 95% probability interval (0.52, 0.60).

The model in this section is defined at the individual level. To aggregate model‐based estimates to the population level, we used poststratification (see Appendix A of the Supporting Information for details). Population counts of age, education, and region of residence for Ugandan women of reproductive age were obtained from the 2014 Uganda Census (Uganda Bureau of Statistics 2016). Posterior estimates of the probability of abortion for each age group/educational level/region combination were then weighted according to their relative weight in the population and aggregated to the national/population level as well as for each age group, education, and region.

#### Computations

The model was fit using the probabilistic programming language Stan (Stan Development Team 2022b) via the rstan R package (Stan Development Team 2022a). Posterior samples were obtained using four parallel chains of 4000 sampling iterations, 2000 of which were warmup iterations. Standard Markov Chain Monte Carlo (MCMC) diagnostics to monitor mixing of the chains were performed. In particular, $\hat{R}$ and the effective sample size were checked.

### Results

Based on the model specified in the previous section, we estimate the overall posterior probability/prevalence of lifetime abortion in Uganda to be 0.209 with a 95% credible interval of (0.134, 0.298). To understand how our results compare to other measures of calculating abortion prevalence, we also calculated the raw (unadjusted) sample proportion of reported confidante abortions along with a poststratified version of the sample proportion. The unadjusted sample proportion of lifetime abortion in Uganda is estimated to be 0.14 with a 95% confidence interval of (0.127, 0.153), whereas the poststratified point estimate is 0.116 with a 95% confidence interval of (0.096, 0.136).^5^ Additionally, we compute a version of the sample proportion adjusted for visibility of abortion (or transmission bias) as done in Giorgio, Sully, and Chiu (2021). The transmission bias‐adjusted point estimate of lifetime abortion is 0.281 with a 95% confidence interval of (0.264, 0.297)^6^ (see Table 3). This estimate was not corrected to account for the nonrepresentativeness of the surrogate sample.

**TABLE 3:** Estimates of the overall prevalence of lifetime abortion, along with 95% uncertainty intervals

Estimate	95% Interval	Method
0.140	(0.127, 0.153)	Unadjusted Proportion
0.116	(0.096, 0.136)	Poststratified
0.209	(0.134, 0.298)	Model‐based
0.281	(0.264, 0.297)	Transmission bias‐adjusted

**FIGURE 2:**
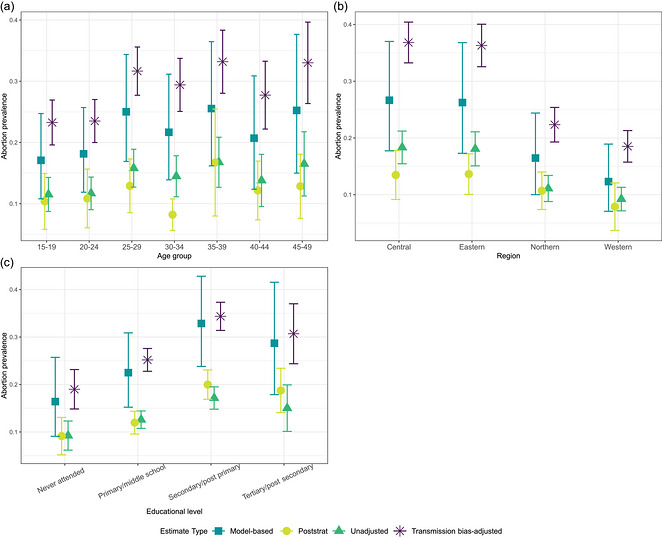
Model‐based posterior estimates of the prevalence of lifetime abortion in Uganda, along with the unadjusted and post‐stratified estimated sample proportions as well as transmission bias‐adjusted estimates. Estimates are plotted along with 95% uncertainty intervals by age, region, and education level

We note that our model‐based point estimate is higher than either the sample estimate or the poststratified sample estimate, while lower than the visibility‐/transmission bias‐adjusted estimate. Although the model‐based estimate has considerable uncertainty associated with it, and drawing any definitive conclusions would not be entirely prudent, we find the results here somewhat encouraging. We expect the unadjusted sample proportions of abortion in the confidante sample to still be an underestimate of the true abortion prevalence. By accounting for the sensitivity and specificity of the instrument in our model, we allow for an upward adjustment of the estimated prevalence. Similar patterns to the overall estimate were observed in the estimates by available sociodemographic characteristics. Figure 2 displays these results broken down by age group (panel a), region (panel b), and educational level (panel c).

The differences between our model estimates and the transmission bias‐adjusted point estimates, however, are likely to be driven by the choice of prior distributions, particularly the prior for SE. The transmission bias adjustment approach suggested in Giorgio, Sully, and Chiu (2021) entails adjusting each numbered confidante sample separately using the proportion of respondents that reported sharing their abortion with confidante 1, 2, or 3. In the Uganda data, the proportion of respondents that reported sharing their abortion experience with each confidante (1, 2, or 3) was similar and on average estimated to be roughly 50%. Assuming a reciprocal relationship in the respondent–confidante dyad, this suggests that roughly half of confidantes' abortions were not visible to the respondents, effectively assuming a lower overall sensitivity of the instrument and leading to a greater inflation of the final transmission bias‐adjusted estimates compared to our model. We wish to emphasize this point. Although our model can easily accommodate additional sources of data to inform the SE and SP parameters, and can easily allow for these parameters to themselves be modeled, in the absence of any auxiliary information, the assumptions built into the prior distributions can have an impact on the final estimates. In Appendix B of the Supporting Information, we further explore the degree to which model estimates are sensitive to choices of priors. We find that, indeed, the choice of prior distributions can meaningfully impact final estimates. Additionally, encoding weaker prior knowledge and less certainty regarding these parameters results in considerably more uncertain estimates.

Moreover, given that no gold‐standard data on abortion are available for Uganda, we cannot reliably validate our model estimates. In efforts to understand the performance of our model, we ran a small simulation study (see Appendix C of the Supporting Information for more details), where we examined how well the model recovers the truth under different scenarios. We apply the specific model we describe in part in the Model section (and in more detail in Appendix A.2 of the Supporting Information) to several hypothetical scenarios where we know the true abortion prevalence and vary the sensitivity and specificity of the survey instrument. Our simulation results suggest that when SE is held fixed and independent of the measured characteristics of the confidantes or their respondents, and the value is set well below 0.5, which is a significant departure from what is assumed about SE in our model, the model has difficulty in recovering the truth. For instance, applying the above model to a scenario where we assume that there is only 25% chance that we observe a given confidante's abortion, meaning fixing SE at 0.25, the model—which by design assumes SE to be much higher and dependent on the abortion status of the confidante's respondent—fails to adequately adjust the estimates upwards and fails to recover the truth. In fact, we find that when we fix SE at values roughly 15% above or below the prior implied values for this parameter, the model struggles to recover the truth particularly well.

## CONCLUSION AND DISCUSSION

Estimating the prevalence and incidence of induced abortion remains a challenging task in abortion research. Measuring abortion is, nevertheless, important, as accurate estimates play a crucial role in informing policies and initiatives around women's sexual and reproductive health. This is true in both high‐income and low‐ and middle‐income countries.

In this paper, we described a statistical framework that offers a different way of conceptualizing biases that arise from violations in the confidante method's key assumptions by linking them to the concepts of sensitivity and specificity of a survey instrument. In most situations, we are unlikely to have perfect knowledge of the sensitivity and specificity of a survey instrument. Rather, our understanding of the possible values of these parameters is likely to vary widely depending on the context. The general Bayesian modeling framework we propose allows us to integrate differing levels of knowledge about the sensitivity, specificity, and potential misclassifications, and incorporate demographic characteristics of the confidantes in the model of the true abortion probability. In addition, such a model affords us a more principled way of incorporating sources of uncertainty and propagating them through the final estimates.

As an illustration, we applied the model to data from an application of the confidante method in Uganda in 2018. We note that in the Ugandan setting, there was only very limited prior information to inform the levels of sensitivity and specificity of the survey instrument or the rate of misclassification, which, unfortunately, were not easily applicable to our case. The posterior estimates for these parameters were driven by the prior we specified. However, the available data did seem to suggest a certain mechanism of differing misclassification on the basis of the abortion experience of the respondents. Given the specification of the model, this information was easily incorporated and encoded in the prior distribution for the sensitivity. Although the model resulted in posterior estimates with wide posterior credible intervals, the point estimates were in a reasonable range, meaning estimates were higher than what would have been obtained directly from the survey on confidantes.

The model we fit to the Uganda data is relatively simple and can easily be extended to incorporate different model specifications or account for additional sources of data, including data of varied quality. The modeling framework is not restricted to a single country and can incorporate multiple countries and time periods, if such data existed. Furthermore, the specificity and sensitivity parameters can themselves be estimated using data, if any were available, and can more explicitly be linked to various biases if rich enough information or measures reasonably approximating those biases can be obtained. In the case of our application in Uganda, no such auxiliary data were available. This meant that even though our model is quite flexible, the estimates were considerably influenced by the assumptions embedded in the prior distribution for SE and SP.

A significant challenge with our approach, and, indeed, most other approaches to measuring abortion, is the absence of gold standard data to validate the resulting estimates. As part of this work, we carried out simulation studies to better understand the limitations of our modeling framework. As we have noted in the Results section, making prior assumptions about the sensitivity and specificity parameters in the model that deviate considerably from some underlying truth about these parameters can have a significant impact on the final estimates. This, while an issue for our framework, is also related to a broader challenge of correctly quantifying certain biases that arise with the confidante method, such as, for instance, biases tied to the visibility of abortion in various contexts.

In the application of our framework, we focused on abortion prevalence. Nevertheless, our approach can be applied just as well to annual abortion incidence, which is often of greater interest in abortion research. We have included the model estimates of the annual incidence rate for 2017 for the Ugandan sample in Appendix E of the Supporting Information. We note that the findings were consistent with our analysis of the overall prevalence of abortion in Uganda. When considering annual incidence, however, it is worth bearing in mind that for some subpopulations, abortions may be quite rare, in which case estimates produced using logistic regression can be biased (see Anderson and Richardson 1979; Albert and Anderson 1984; Santner and Duffy 1986; McCullagh and Nelder 1989; Lesaffre and Albert 1989). This is particularly true for maximum‐likelihood‐based estimates, but can be true in the Bayesian setting as well. In such cases, it is recommended that proposed remedies are implemented, which for Bayesian models involve a careful consideration of prior distributions (for a more classical discussion of biases and remedies, see Firth 1993; Heinze and Schemper 2002; Greenland, Mansournia, and Altman 2016; for considerations of priors in the Bayesian setting see Gelman et al. 2008; Hamra, MacLehose, and Cole 2013; Greenland and Mansournia 2015).

The approach we describe in this paper has a number of shortcomings that we could not address. First, while we provided a detailed discussion of the key assumptions that underlie the confidante method, the modeling approach we describe is not equipped to adjust for all sources of bias. In particular, the model does not explicitly adjust for certain biases arising from violations in assumptions associated with the denominator, namely we cannot fully account for the unrepresentativeness of the surrogate sample, beyond what can be remedied with a poststratification step. The method makes a somewhat strong assumption that the respondents who report on any confidantes do not systematically differ from those who report none. However, it is likely that women who are socially more marginalized are missing from our sample of confidantes. If their abortion experiences differ from those of their more socially connected peers than the resulting prevalence estimates would be biased, regardless of how well we can account for the survey instrument's sensitivity and specificity. We make an additional assumption that among women who report at least one confidante, those with larger circles of confidantes do not differ in any systematic ways along unmeasured characteristics from those with smaller networks. If the abortion experiences and the propensity to report on those experiences also differ among these groups of women, the abortion prevalence estimates might be unduly biased. While not directly adjusting for violations in the assumption surrounding the denominator (in particular, the presence of barrier effects and popularity bias) is a significant limitation for our estimation approach, this is also a more fundamental issue and largely an existing drawback of most TPR methods to measure abortion, including the confidante method. Without further research on the differences in abortion experiences among relevant groups of women (e.g., among socially more connected and more marginalized women), any model‐based adjustments would require strong assumptions. Second, for the purposes of this work, we make the assumption that there is no multiplicity in the sample. Despite the fact that researchers' definition of a confidante places no restrictions on geographical location, given the somewhat larger number of households selected to participate in the survey within each enumeration area, it is possible that some women might appear in the surrogate sample more than once. In principle, this is not likely to bias the estimates, however it might impact their precision. A difficulty arises in the case where several respondents report on the same confidante, but their knowledge of that confidante's abortion experience is flawed.

As a way of concluding, we wish to emphasize that the main goal of this paper was not to necessarily produce improved estimates of the prevalence of abortion, but rather to offer an entirely different tool for conceptualizing biases that arise in typical applications of the confidante method. Even though our estimates have significant uncertainty associated with them, we have offered a principled statistical way of handling uncertainty and propagating it through to the final estimates. Additionally, while in our specific application we did not have supplementary data on SE and SP of the survey instrument, in other contexts or for other indicators there might be auxiliary information that can be used to inform the misclassification in the outcomes. Finally, our goal was to offer a statistical modeling approach where adjustments for some of the biases arising from the confidante method can be made in a more uniform and integrated manner. Significant work around such bias adjustments already exists in the literature; however, that work has been somewhat unsystematic and often lacking in its treatments of uncertainty.

## Supporting information

Data S1

## Data Availability

All data supporting the findings in this paper are publicly available at https://doi.org/10.34976/d41k‐sh61

## Footnotes

1 Here we mean the numerator or denominator of a classical abortion incidence or prevalence, where the numerator is the number of reported abortions and the denominator the size of the relevant analytic sample.

2 The data are publicly available and accessible at https://doi.org/10.34976/MEQV‐3S60.

3 The full female questionnaire administered as part of the survey can be downloaded at https://doi.org/10.34976/MEQV‐3S60 or directly accessed at https://www.pmadata.org/sites/default/files/2019‐11/PMA2018_UGR6‐Female‐Questionnaire.pdf

4 The estimated proportion here was adjusted to account for the complex survey design

5 Poststratified estimates and standard errors were obtained using the survey package in R (Lumley 2020).

6 Note that the 95% confidence interval for the transmission bias‐adjusted estimate is based on a crude approximation of the standard error of the adjusted, or more specifically inflated, proportion. The true standard error is likely greater.
