# Supplementary material for: A Bayesian Framework to Account for Misclassification Error and Uncertainty in the Estimation of Abortion Prevalence
Source: Stud Fam Plann. 2026 May 5;57(2):234–55. doi: 10.1111/sifp.70053 (PMC13275040; doi:10.1111/sifp.70053)
Supplement: Supplementary file 1 — Data S1 [file SIFP-57-234-s001.pdf]

Appendix: Supplemental Materials

A Technical Details

A.1 Modeling framework

We being by setting notation and laying out relevant results. Let $Y_i^{\texttt{TRUE}}$ denote the true, but possibly unobservable abortion status for individual i

$$Y_i^{\texttt{TRUE}} = \begin{cases} 1 & \text{if person } i \text{ has had an abortion} \\ 0 & \text{otherwise} \end{cases}$$

and similarly let $Y_i^{\texttt{OBS}}$

$$Y_i^{\texttt{OBS}} = \begin{cases} 1 & \text{if person } i \textbf{ reports/is reported as} \text{ having had an abortion} \\ 0 & \text{otherwise} \end{cases}$$

denote the observed, or more specifically, reported abortion status for individual i. $Y_i^{\texttt{OBS}}$ here is the data we obtain through the survey and is one of two values, ***yes*** or ***no***, coded as 1 or 0.

We denote the probability of true abortion for an individual i as

$$\gamma_i^{\texttt{TRUE}} = \mathrm{P}(Y_i^{\texttt{TRUE}} = 1)$$

and, similarly, the probability that a specific individual i reports/is reported as having had an abortion as

$$\gamma_i^{\texttt{OBS}} = \mathrm{P}(Y_i^{\texttt{OBS}} = 1).$$

Since we do not have the ability to observe the true underlying abortion status for each individual i, we assume $Y_i^{\texttt{OBS}} \sim \text{Bernoulli}(\gamma_i^{\texttt{OBS}})$

We define the sensitivity (**SE**) of the instrument as the probability of observing a true underlying abortion, or the true positive rate. More formally, the sensitivity can be expressed as

$$\mathrm{SE} = \mathrm{P}(Y_i^{\texttt{OBS}} = 1 \mid Y_i^{\texttt{TRUE}} = 1) \quad \text{for all } i$$

Similarly, we define the specificity (**SP**), which is the probability that the instrument correctly captures a true underlying absence of abortion or the true negative rate, as

$$\text{SP} = \text{P}(Y_i^{\texttt{OBS}} = 0 \mid Y_i^{\texttt{TRUE}} = 0) \quad \text{for all } i$$

The observed and true probability of abortion can then be related in the following way:

$$\begin{aligned}
\gamma_i^{\texttt{OBS}} &= \text{P}(Y_i^{\texttt{OBS}} = 1) \\
&= \text{P}(Y_i^{\texttt{OBS}} = 1 \mid Y_i^{\texttt{TRUE}} = 1)\text{P}(Y_i^{\texttt{TRUE}} = 1) + \text{P}(Y_i^{\texttt{OBS}} = 1 \mid Y_i^{\texttt{TRUE}} = 0)\text{P}(Y_i^{\texttt{TRUE}} = 0) \qquad \text{(A1)} \\
&= \text{SE}\ \gamma_i^{\texttt{TRUE}} + (1 - \text{SP})(1 - \gamma_i^{\texttt{TRUE}})
\end{aligned}$$

The true abortion probability then can be expressed as a function of what we observe, along with the SE and SP, as follows:

$$\gamma_i^{\texttt{TRUE}} = \frac{\gamma_i^{\texttt{OBS}} - (1 - \text{SP})}{\text{SE} - (1 - \text{SP})} \qquad \text{(A2)}$$

Differential misclassification

The expression in (A2) assumes that SE and SP are constant across characteristics of the individual, i.e. that misclassification occurs *nondifferentially.* In the abortion context it is likely that misclassification varies by factors other than the underlying abortion truth, i.e. that responses are misclassified *differentially.*

Let $\mathbf{S}$ denote a specific covariate or a combination of covariates, where $\mathbf{S} = s$ represents a specific stratum, s, of that covariate.

We specify the sensitivity which varies by strata of $\mathbf{S}$ as

$$\text{SE}_s = \text{P}(Y_i^{\texttt{OBS}} = 1 \mid Y_i^{\texttt{TRUE}} = 1, \mathbf{S}_i = s)$$

and similarly for specificity

$$\text{SP}_s = \text{P}(Y_i^{\texttt{OBS}} = 0 \mid Y_i^{\texttt{TRUE}} = 0, \mathbf{S}_i = s),$$

where values of sensitivity and specificity are now indexed by strata s. Then, we can define $\gamma_i^{\texttt{TRUE}}$ separately

for each stratum of the covariate $\mathbf{S}$ as follows:

$$\gamma^{\texttt{TRUE}}_{i|\mathbf{S}_i=s} = \frac{\gamma^{\texttt{OBS}}_{i|\mathbf{S}_i=s} - (1 - \mathrm{SP}_s)}{\mathrm{SE}_s - (1 - \mathrm{SP}_s)} \tag{A3}$$

General model

Mathematical description of a more general model is given below:

$$Y_i^{\texttt{ OBS}} \mid \gamma_i^{\texttt{OBS}} \sim \text{Bernoulli}\left(\gamma_i^{\texttt{OBS}}\right) \tag{A4}$$

$$\gamma_i^{\texttt{OBS}} = \mathrm{SE}_{\mathbf{S}(i)}\ \gamma_i^{\texttt{TRUE}} + (1 - \mathrm{SP}_{\mathbf{S}(i)})(1 - \gamma_i^{\texttt{TRUE}}) \tag{A5}$$

$$\gamma_i^{\texttt{TRUE}} = g^{-1}\left(\mathbf{X}\beta\right) \tag{A6}$$

where $Y_i^{\texttt{ OBS}}$, $\gamma_i^{\texttt{OBS}}$, and $\gamma_i^{\texttt{TRUE}}$ are as defined earlier in this section. Equation (A6) describes our understanding of the process that guides the true probability of having had an abortion, $\gamma_i^{\texttt{TRUE}}$. Here $g(\cdot)$ denotes a monotone link function which allows us to relate the probability of interest, $\gamma_i^{\texttt{TRUE}}$, to various socio-demographic characteristics captured in $\mathbf{X}\beta$.

Specifying prior distributions for SE and SP

Since both SE and SP are probabilities, one possible choice for modelling uncertainty about them would be through a beta distribution, Beta(η, ν) — a continuous probability distribution defined on the interval $[0, 1]$, where the parameters η and ν control the shape of the distribution. The beta family of distributions is considered suitable for modelling proportions (Gelman et al. 2013). An appropriate choice of Beta(η, ν) as prior distribution for either SE or SP can be made by specifying reasonable values for some of the distributions parameters. For instance, one may specify a prior mode, the most frequent value, along with a value for a specific percentile such that a prior probability interval with desired width could be obtained. When considering appropriate values for prior quantities, in particular an adequate value for the prior mode, it is helpful to note that the mode of a beta distribution, Beta(η, ν), can also be thought of as $\eta - 1$ successes out of $\eta + \nu - 2$ trials in a binomial experiment. For example, in the abortion context, based on expert knowledge we might believe that a survey instrument has relatively high specificity and that it is reasonable to assume that the survey instrument likely correctly identifies those that have not had an abortion in 98 out of 100 cases. This would suggest that we expect our mode to be around 0.98. Suppose also that we are willing to place no more than 0.025 prior mass on values less than 0.9. In this case, one example of a Beta distribution satisfying these requirements is a Beta(49, 2).

Obtaining population-level estimates

Population-level estimates are obtained from model-based estimates through poststratification: a commonly used survey sampling technique for weighting non-representative samples to produce more accurate estimates of population quantities. Poststratification requires that we know the joint distribution of the population over the individual characteristics on which we are poststratifying (typically these are the predictors in our model). To obtain a population-level estimate of the probability/prevalence of abortion, we first use the individual-level model to estimate the probability of having an abortion for each subgroup or possible combination of covariates specified in $\mathbf{X}\beta$. We then aggregate these estimates up to the national level by appropriately weighting them by the relative proportion of each of these subgroups in the population. More formally, letting j be one of $1, \ldots, J$ subgroups that a confidante could belong to (again, based on characteristics we are interested in poststratifying on), and N_j denote the population size for the j-th subgroup, then the population-level estimate of the true probability of abortion can be obtained as $\hat{\gamma}^{\texttt{TRUE}} = \frac{\sum_j N_j \hat{\gamma}_j^{\texttt{TRUE}}}{\sum_j N_j}$.

A.2 Full model specification for the Uganda case study

$$Y_i^{\texttt{OBS}} \mid \gamma_i^{\texttt{OBS}} \sim \text{Bernoulli}\left(\gamma_i^{\texttt{OBS}}\right) \tag{A7}$$

$$\gamma_i^{\texttt{OBS}} = \gamma_i^{\texttt{TRUE}}\, \text{SE}_{\mathbf{S}(i)=s} + (1-\text{SP})(1-\gamma_i^{\texttt{TRUE}}) \tag{A8}$$

$$\gamma_i^{\texttt{TRUE}} = \text{logit}^{-1}\left(\beta_0 + \mathbf{X}_{\text{age},i}\, \boldsymbol{\beta}^{\text{ age}} + \mathbf{X}_{\text{edu},i}\, \boldsymbol{\beta}^{\text{ edu}} + \mathbf{X}_{\text{region},i}\, \boldsymbol{\beta}^{\text{ region}}\right) \tag{A9}$$

$$\text{SP} \sim \text{Beta}\,(200, 1) \tag{A10}$$

$$\text{SE}_{\ S(i)=1} \sim \text{Beta}\,(542, 242) \tag{A11}$$

$$\text{SE}_{\ S(i)=0} \sim \text{Beta}\,(250, 200) \tag{A12}$$

$$\beta_0 \sim \text{N}(0, 3) \tag{A13}$$

$$\beta_a^{\text{age}} \sim \text{N}(0, 2) \text{ for each } a \tag{A14}$$

$$\text{where } a \in \{\text{20-24, 25-29, 30-34, 35-39, 40-44, 45-49}\}$$

$$\beta_e^{\text{edu}} \sim \text{N}(0, 2) \text{ for each } e \tag{A15}$$

$$\text{where } e \in \{\text{never attended, tertiary/post secondary, secondary/post primary}\}$$

$$\beta_r^{\text{region}} \sim \text{N}(0, 2) \text{ for each } r \tag{A16}$$

$$\text{where } r \in \{\text{central, northern, western}\}$$

In this application of the model, the outcome of interest is whether or not a confidante has ever had an

abortion. The model quantities are largely as defined in the general model described in (A4) - (A6). Here, however, we impose specific conditions on the SE and SP parameters. We assume SP remains constant across characteristics of the confidantes, SE depends on the abortion status of each confidante's respondent, meaning SE is indexed by $\mathbf{S}(i) = s$, such that $\mathbf{S}(i) = 0$ if confidante i's respondent did not report an abortion, and $\mathbf{S}(i) = 1$ if confidante i's respondent reported an abortion. Equation (A10) gives the prior distribution for SP, while (A11) and (A12) give the priors for the two SE parameters. $\text{logit}^{-1}()$ refers to the inverse logit function, defined as $\text{logit}^{-1}(x) = \frac{1}{1+e^{-x}}$. In this logistic regression specification, the overall model intercept, β_0, is assigned a $\text{N}(0, 3)$ prior, while each component of $\boldsymbol{\beta}^{\text{age}}$, $\boldsymbol{\beta}^{\text{edu}}$, $\boldsymbol{\beta}^{\text{region}}$ is given a $\text{N}(0, 2)$ prior. Such prior distributions are considered weakly informative (Gelman et al. 2013).

B Model estimates' sensitivity to choices of priors

The model estimates presented in Section 4.3 relied on priors for the Sensitivity and Specificity parameters that were informed by specific features of the data. However, as we have noted earlier the parameters are largely informed by the prior distributions. Here we illustrate the degree to which model estimates are sensitive to choices of priors for the Sensitivity and Specificity parameters. Recall that in the model specification provided in Section 4.2 we assume that the sensitivity of the survey instrument depends on the abortion status of the respondent (a 0/1 variable $S(i)$, where, $S(i) = 1$ refers to respondents reporting having had an abortion, and $S(i) = 0$ not). Specifically, we assumed the following prior for the sensitivity and specificity of the instrument: $\text{SE}_{S(i)=0} \sim \text{Beta}(250, 200)$, $\text{SE}_{S(i)=1} \sim \text{Beta}(542, 242)$, $\text{SP} \sim \text{Beta}(200, 1)$.

We examine the model estimates under the following combinations of prior specifications:

- **Case 1**: Original priors, namely $\text{SP} \sim \text{Beta}(200, 1)$ which results in a prior mode for SP of 1 and 95% prior probability interval of (0.98,1); $\text{SE}_{S(i)=0} \sim \text{Beta}(250, 200)$, with a prior mode of 0.56 and 95% interval (0.51, 0.60); and finally $\text{SE}_{S(i)=1} \sim \text{Beta}(542, 242)$, with a prior mode of 0.69 and 95% prior probability (0.66, 0.72).
- **Case 2**: Allowing for wider prior probability intervals for the two SE parameters. The priors for SE are centered at the original prior modes (as in Case 1), but we allow for greater uncertainty in our prior knowledge, more specifically $\text{SE}_{S(i)=0} \sim \text{Beta}(20, 16)$ with prior mode at 0.56 and 95% prior probability interval (0.39,0.71) ; $\text{SE}_{S(i)=1} \sim \text{Beta}(23, 10)$ with prior mode at 0.7 and 95% prior probability (0.65,0.84); Prior for SP remains as in Case 1, $\text{SP} \sim \text{Beta}(200, 1)$.
- **Case 3**: Allowing for wider prior probability interval for SP, while maintaining the original priors for SE. Specifically, $\text{SP} \sim \text{Beta}(31, 1)$ with mode at 1 and 95% prior probability interval (0.88,1). Priors for SE as in Case 1.
- **Case 4**: Allowing for wider prior probability for both SE and SP. Modes of each prior distribution held at the same value as in Case 1; $\text{SP} \sim \text{Beta}(31, 1)$ with mode at 1 and 95% prior probability interval (0.88,1); $\text{SE}_{S(i)=0} \sim \text{Beta}(20, 16)$ with prior mode at 0.56 and 95% prior probability interval (0.39,0.71) ; $\text{SE}_{S(i)=1} \sim \text{Beta}(23, 10)$ with prior mode at 0.7 and 95% prior probability (0.65,0.84);
- **Case 5**: Allowing for lower prior modes for the SE parameters, while maintaining a relatively tight prior probability intervals, with $\text{SE}_{S(i)=0} \sim \text{Beta}(200, 470)$ with prior mode at 0.3 and 95% prior probability interval (0.26,0.33); $\text{SE}_{S(i)=1} \sim \text{Beta}(250, 200)$ with prior mode at 0.56 and 95% prior probability (0.51,0.6); and $\text{SP} \sim \text{Beta}(200, 1)$ with a prior mode of 1 and 95% prior probability interval of (0.98,1).
- **Case 6**: Lower prior mode for SP, while prior mode and uncertainty for SE left as in Case 1.SP $\sim$

$\text{Beta}(300, 10)$ with a prior mode of 0.97 and prior probability interval of (0.95, 0.98);SE $_{S(i)=0} \sim \text{Beta}(250, 200)$, with a prior mode of 0.56 and 95% interval (0.51, 0.60); and finally SE $_{S(i)=1} \sim \text{Beta}(542, 242)$, with a prior mode of 0.69 and 95% prior probability (0.66, 0.72).

- **Case 7**: Allowing for lower modes for all three parameters, while keeping the prior probability intervals somewhat tight. SE $_{S(i)=0} \sim \text{Beta}(200, 470)$ with prior mode at 0.3 and 95% prior probability interval (0.26,0.33); SE $_{S(i)=1} \sim \text{Beta}(250, 200)$ with prior mode at 0.56 and 95% prior probability (0.51,0.6);and SP $\sim \text{Beta}(200, 1)$ with a prior mode of 1 and 95% prior probability interval of (0.98,1).
- **Case 8**: Allowing for lower modes for all three parameters, with a little more uncertainty around them. SE $_{S(i)=0} \sim \text{Beta}(35, 80)$ with prior mode at 0.3 and 95% prior probability interval (0.22,0.39); SE $_{S(i)=1} \sim \text{Beta}(60, 60)$ with prior mode at 0.5 and 95% prior probability (0.41,0.59);and SP $\sim \text{Beta}(50, 10)$ with a prior mode of 0.84 and 95% prior probability interval of (0.73,0.92).
- **Case 9**: Allowing for lower modes for all three parameters, with more uncertainty around them. SE $_{S(i)=0} \sim \text{Beta}(5, 10)$ with prior mode around 0.3 and 95% prior probability interval (0.13,0.58); SE $_{S(i)=1} \sim \text{Beta}(19, 15)$ with prior mode at 0.56 and 95% prior probability (0.39,0.72);and SP $\sim \text{Beta}(25, 5)$ with a prior mode of 0.84 and 95% prior probability interval of (0.65,0.93).
- **Case 10**: SE $\sim \text{Beta}(90, 90)$ with prior mode of 0.5 and prior probability interval of (0.43,0.57), and SP $\sim \text{Beta}(105, 3)$ with a prior mode of 0.98 and a prior probability interval of (0.93, 1).
- **Case 11**: SE $\sim \text{Beta}(45, 45)$ with prior mode of 0.5 and prior probability interval of (0.4,0.6), and SP $\sim \text{Beta}(31, 1)$ with a prior mode of 1 and a prior probability interval of (0.88,1).

We note that Cases 1-9 correspond to settings where the prior for SE depends on the abortion status of the respondents — i.e. we set two separate prior distributions — whereas Case 10 and Case 11 assume a single prior probability distribution for the SE parameter.

Figure B1 summarizes the results of our sensitivity analysis. The results point to the fact that the model estimates can, indeed, be sensitive to the choices of prior distribution for SE and SP. Speaking broadly, decreases in prior mode value for SE correspond to generally higher posterior estimates, while decrease in SP have the opposite effect, which is expected behavior. However, beyond those broad patterns, there is a less clear picture about how changes in prior assumptions around the value of the modes or the prior probability intervals affect the final estimates.

Consider Cases 5-7. All three cases here deal with priors whose prior probability intervals are roughly the same width as those in Case 1, but whose modes are lower. In Case 5, lowering the modes for both SE parameters (to 0.3 and 0.5), while keeping SP as is, results in much higher model estimate of abortion prevalence (0.389 with 95% credible interval (0.249,0.551)), but also substantial increases in the uncertainty

around the estimate. Conversely, in Case 6, lowering the mode for SP (from 1 to 0.97) results in lower estimated abortion prevalence (0.17, 95% CI(0.09, 0.26)). Case 7, where all three parameters are assumed to have lower modes, produces estimates somewhere in between Case 5 and 6. This is expected, as assumptions about SE and SP pull the estimates in opposite direction. Cases 2-4 rely on priors whose prior modes are as in original model, but where we consider wider prior probability intervals. As expected, the estimates in all three cases change, as do the widths of the posterior credible intervals, suggesting our certainty around the value of the priors can have a significant impact on the estimates. Cases 8-9 are an illustration of less reasonable prior choices. In both cases we use priors with lower modes and much higher prior probability. Case 9 is a particularly stark example of an unreasonable estimate. We note here that because SE and SP are almost entirely informed by the priors, assuming prior distributions that allow for wide prior probability intervals poses serious computational challenges for the model. In this setting, highly "uninformative" priors — such as Beta$(1, 1)$, which is equivalent to assuming a uniform distribution on the interval $(0, 1)$ — would not be appropriate.

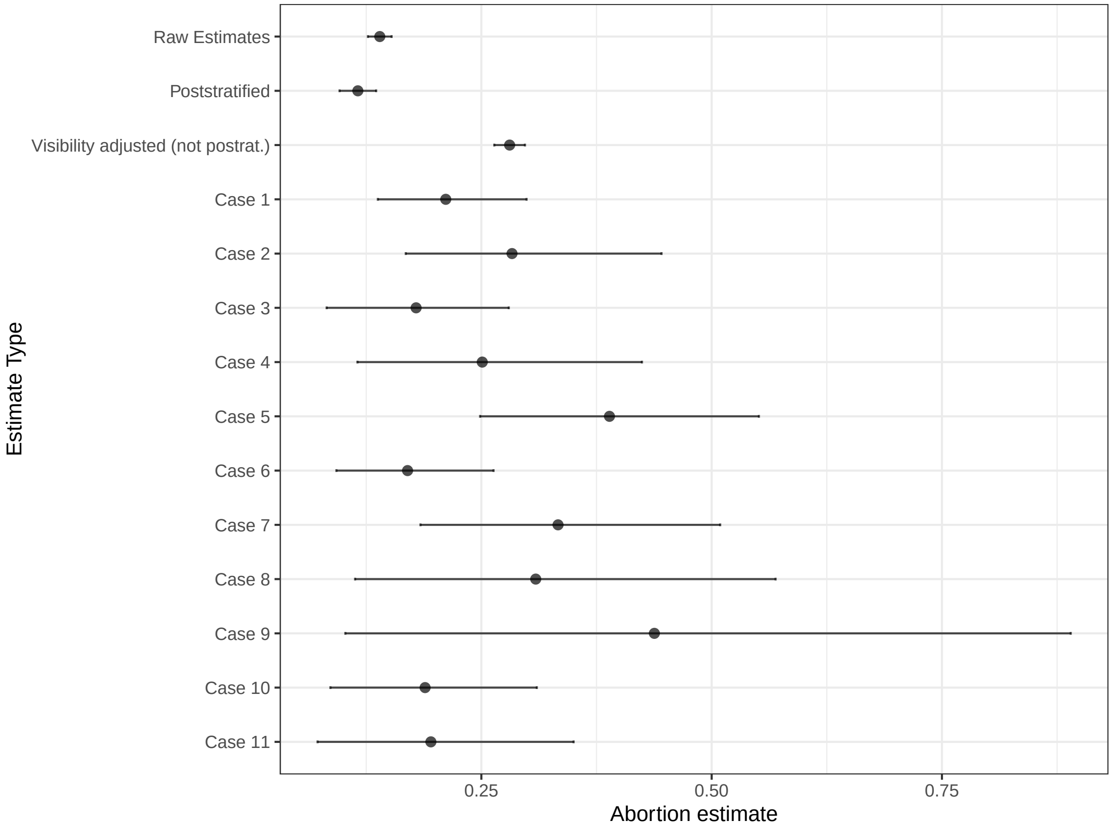

FIGURE B1 Model estimates of abortion prevalence under Case 1 - 11, along with raw sample estimates, poststratified estimates, and visibility adjusted (but not poststratified) estimates. In Case 1 - 11, error bars denote 95% posterior credible intervals; for the rest of the estimates the intervals capture empirical standard errors.

C Simulation study

One of the main limitations of our proposed modelling framework is the absence of gold standard data, meaning that we lack the ability to appropriately validate our model estimates. In this section we carry out a simple simulation study designed to investigate how our model performs under hypothetical scenarios in which we vary SE and SP and have complete knowledge of the true abortion rate.

For simplicity, we generate a set of respondents which produce a nationally representative sample of 2000 confidantes, and we let the true lifetime abortion rate vary by age, education level, and region, such that the probability of ever having had an abortion increases with age and level of education and is the highest in the Central region of Uganda and lowest in the west of the country. The overall true proportion of confidantes who have had an abortion is set at 0.2435.

For the observed abortion outcomes, we consider the following five hypothetical scenarios:

- **Scenario 1**: SE is constant and set at 25%, independent of the characteristics of the confidante or respondent. For each confidante in this scenario there is a 25% chance that we observe her true abortion status.
- **Scenario 2**: SE is constant and set at 75%.
- **Scenario 3**: SE is fixed at 50% and 1-SP is set at 5%. Neither one of the parameters vary with the characteristics of the confidantes.
- **Scenario 4**: SE varies by the age of the confidante, where it is lowest for confidantes aged 15-19 and set at 25% and highest among those aged 35-39 and set at 93%.
- **Scenario 5**: SE varies by the respondent abortion status and the rank of the confidante (one of 1,2,3), such that SE is highest among first confidantes whose respondents reported an abortion and set at around 80% and lowest among the third confidantes of those respondents that did not report an abortion and set at around 20%.

We simulate an observed abortion response for each confidante under each of the five scenarios. We repeat the experiment 100 times. For each such simulated data set we fit the exact model outlined in Section 4.2 and investigate how well the model does in recovering the true prevalence. We do this in part to understand what happens in cases where we make wrong assumptions about the mechanism of underreporting.

Figure C1 below summarizes our findings. In cases where SE is fixed and set well above or bellow the values assumed in the priors of our original model, and no other demographic characteristics determine the SE values, the model struggles to recover the truth. This is in line with what we expect. Recall that in our

model we make a prior assumption that SE is around 0.5 for confidantes whose respondents did not report an abortion, and around 0.7 for the fewer confidantes whose respondents did report an abortion. Under Scenario 1 (first panel of Figure C1) the model fails to adequately adjust the estimates upward as it operates under the assumption that the SE is much higher. The other model component cannot sufficiently remedy the situation either, as under both Scenario 1 and 2, with a fixed probability (independent of confidantes' characteristics) we do not observe some of the true abortions. In Scenario 2, conversely, the model overestimates the true prevalence. By contrast, in Scenario 3, SE, though still fixed, is more in line with the prior assumptions of the model and as a consequence the model estimates are considerably closer to the true prevalence. However, the model in this case is likely inadequately accounting for the larger false positive rate, and though closer to the true prevalence the model estimates are still somewhat inflated.

Scenarios 4 and 5 are a bit more interesting. Here SE varies by characteristics of the confidante or the respondent. In both these cases, the assumptions about SE likely drive the estimates to a large part (note that the overall SE in both Scenario 4 and 5 sits at around 0.5), however it is possible that the other modelling components (accounting for age in the process model or differentiating between respondents' abortion status in the SE values), are aiding in bringing the estimates closer to the truth.

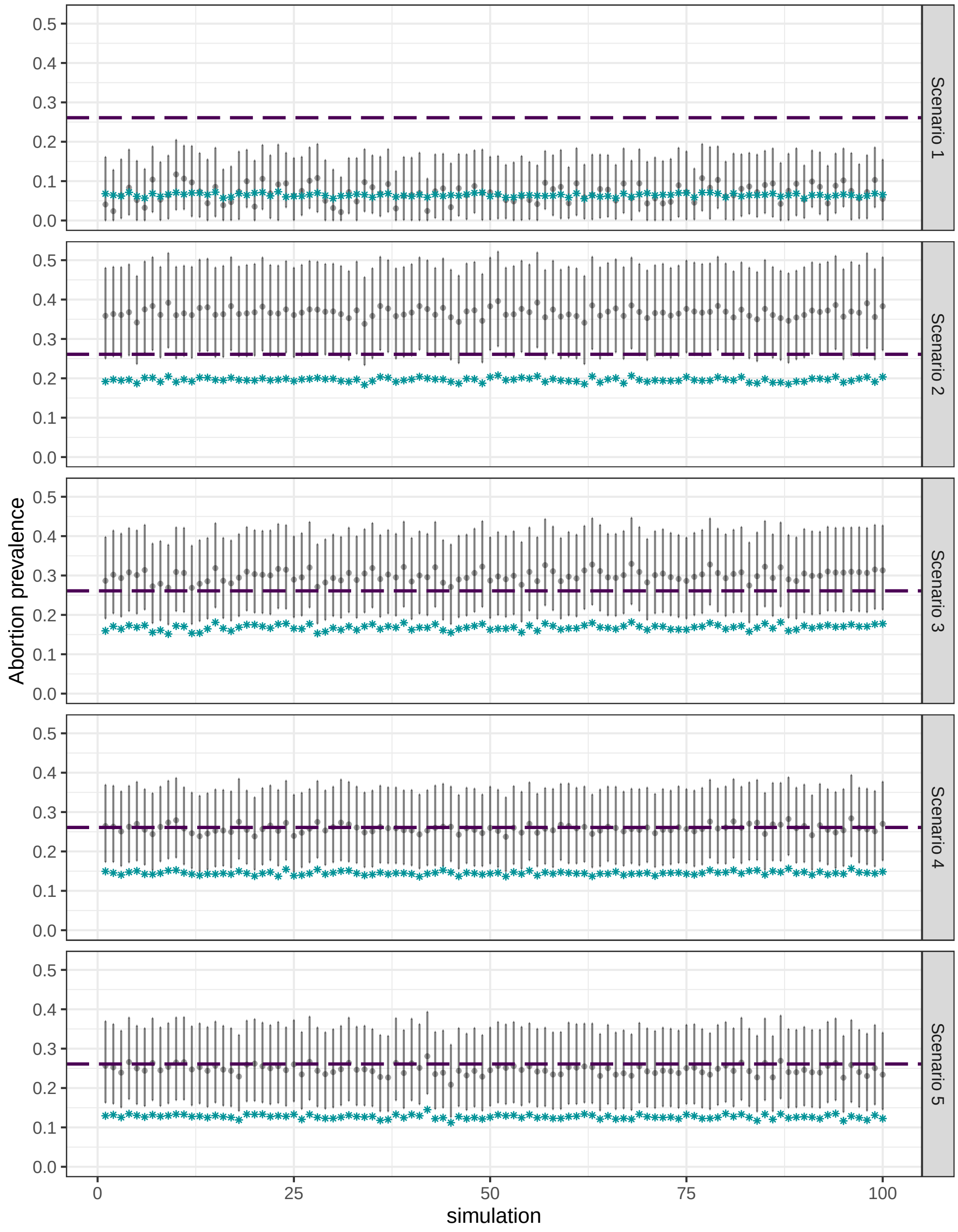

FIGURE C1 Model estimates (black points) along with 95% credible intervals from 100 simulated samples under five different scenarios. The dashed deep purple line represents the true abortion prevalence, while the blue stars represent the observed abortion responses under each of the five scenarios.

D Model evaluation

Plots of the posterior distribution along with prior densities for select number of parameters are presented below (Figure D1). In our application there were no auxiliary data available to us to further help inform the parameters for the sensitivity and specificity (SE $_{S(i)=0}$, SE $_{S(i)=1}$, and SP). These parameters are, therefore, largely informed by the prior distribution we impose. This can be seen in Figure D1. The posterior distributions of SE and SP are exactly what we would expect given the priors we assume.

Figure D2 below plots the prior vs posterior densities for a subset of the coefficient parameters. For the intercept we assumed a $N(0,3)$ prior, while the remaining coefficients associated with the different categories of age, education, and region were given a $N(0,2)$.These are arguably quite wide priors, but as is evident in the plot the model learns well from the data.

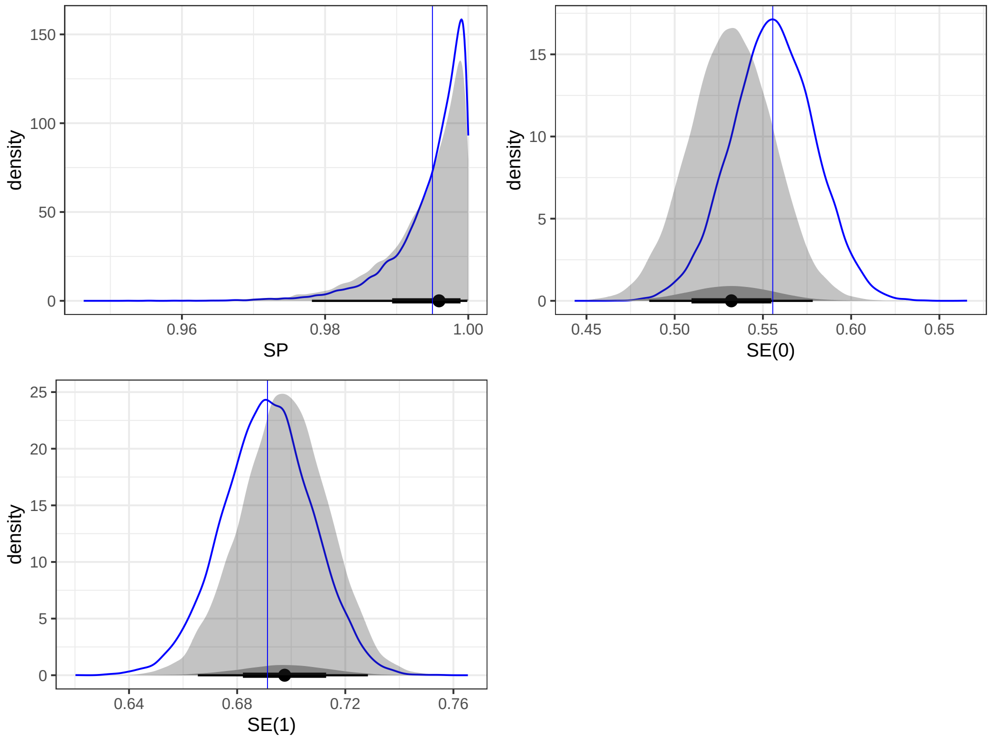

FIGURE D1 Prior and posterior distribution for the sensitivity and specificity parameters. The grey shaded area denotes the posterior distribution of each parameter, along with a marker for the posterior median at the bottom of the density. The blue curve corresponds to the prior density of each parameter.

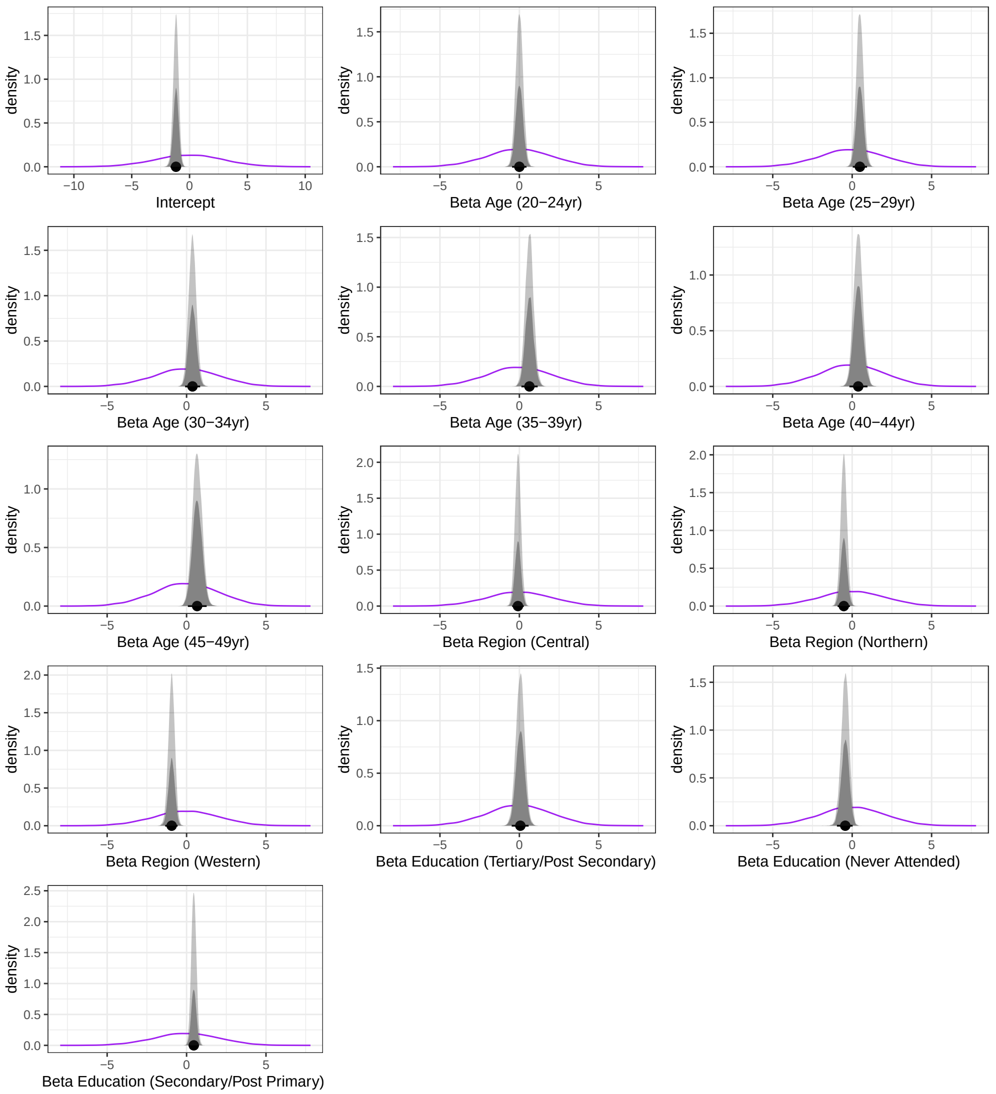

FIGURE D2 Prior and posterior distribution for the regression coefficients. The grey shaded area denotes the posterior distribution of each parameter, along with a marker for the posterior median at the bottom of the density. The purple curve corresponds to the prior density of each parameter.

Prior and Posterior Predictive Checks

Figure D3 provides a summary of the simulated data under the prior distributions specified in the model.

The choices of priors seem to generate relatively plausible data sets with quite a range in proportions.

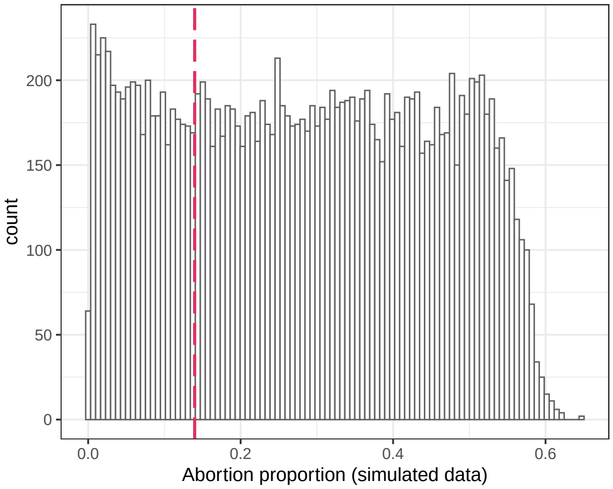

FIGURE D3 Simulated draws from the prior predictive distribution. Red line indicates the observed overall abortion proportion in the Uganda sample.

Figure D4 shows reliability diagrams for the two abortion probability parameters. Reliability diagrams are in essence calibration plots which capture the agreement between posterior predicted probabilities and frequency of observed events in the data — here we present reliability diagrams in the style of Dimitriadis, Gneiting, and Jordan (2021). Note that in the left panel in Figure D4 our estimate of the parameter $\gamma^{\texttt{OBS}}$ seems to be well calibrated, while on the right the plot suggest that the model predicts more instances with higher posterior abortion probability (associated with $\gamma^{\texttt{TRUE}}$) than would be expected in the data, and the red line therefore deviates from the diagonal. This is expected, as in our modeling framework we define $\gamma^{\texttt{TRUE}}$ and $\gamma^{\texttt{OBS}}$ in precisely such a way as to account for the underreporting we suspect exists in the true abortion prevalence, $\gamma^{\texttt{TRUE}}$.

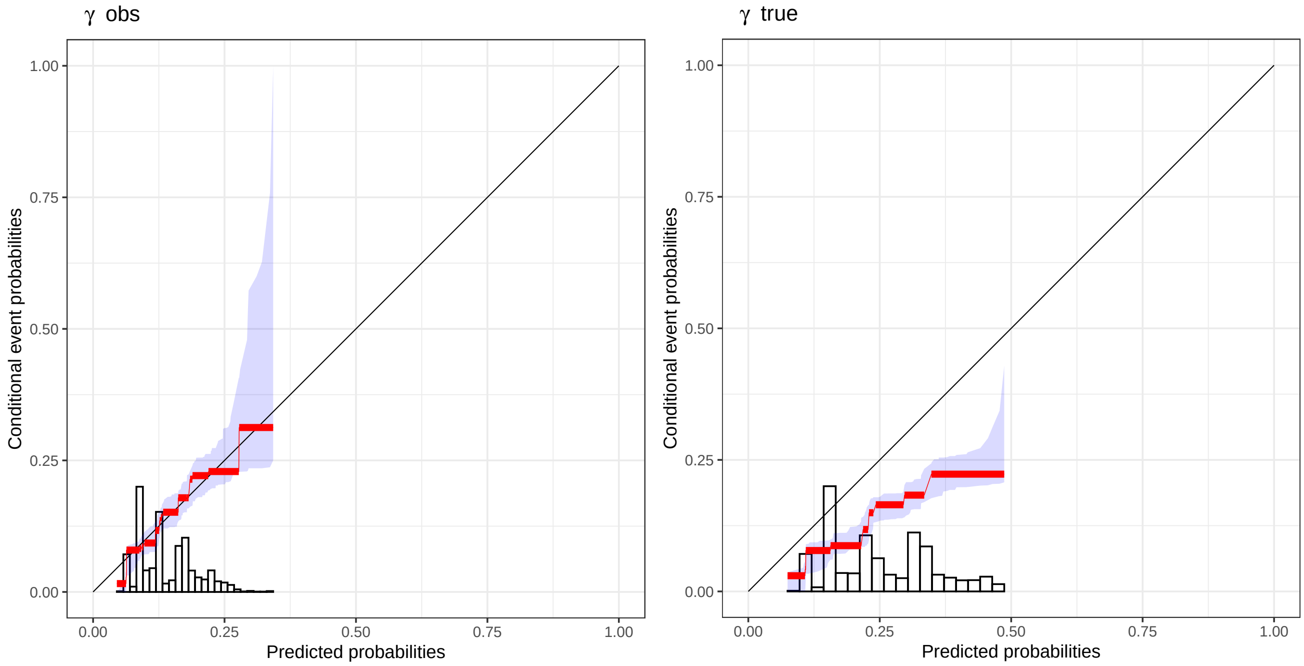

FIGURE D4 Reliability diagrams for the posterior predictions of the true and observed abortion probability parameters. Predicted probabilities are plotted against conditional event probabilities, which give the observed frequency of abortions for a given predicted probability. The shaded blue regions capture the confidence around the estimates.

E Estimates of annual abortion incidence

Estimates of the annual abortion incidence in Uganda for abortions occurring in the year 2017 are presented below. The model specification remains the same as in the estimation of abortion prevalence, which is outlined in Appendix A.2. The mean model specification, as well as assumptions around the SE and SP parameters, including the priors we assign them, remain unchanged. The only departure from the model described in (A7) - (A16) is the slight modification in the prior distributions assigned to the regression coefficients. Namely, we set

$$\beta_0 \sim \mathrm{N}(0,1) \tag{E1}$$

$$\beta_a^{\text{age}} \sim \mathrm{N}(0,1) \text{ for each } a \tag{E2}$$

$$\text{where } a \in \{\text{20-24, 25-29, 30-34, 35-39, 40-44, 45-49}\}$$

$$\beta_e^{\text{edu}} \sim \mathrm{N}(0,1) \text{ for each } e \tag{E3}$$

$$\text{where } e \in \{\text{never attended, tertiary/post secondary, secondary/post primary}\}$$

$$\beta_r^{\text{region}} \sim \mathrm{N}(0,1) \text{ for each } r \tag{E4}$$

$$\text{where } r \in \{\text{central, northern, western}\}$$

Such priors are considered somewhat informative (Gelman et al. 2013).

TABLE E1 Estimates of the annual abortion incidence for Uganda in 2017

Estimate	95% Interval	Method
0.039	(0.032, 0.046)	Unadjusted Proportion
0.049	(0.031, 0.066)	Poststratified
0.057	(0.02, 0.11)	Model-based
0.079	(0.069, 0.089)	Transmission bias-adjusted

References

Dimitriadis, Timo, Tilmann Gneiting, and Alexander I. Jordan. 2021. "Stable Reliability Diagrams for Probabilistic Classifiers." *Proceedings of the National Academy of Sciences* 118 (8): e2016191118. https://doi.org/10.1073/pnas.2016191118.

Gelman, Andrew, John B Carlin, Hal S Stern, David B Dunson, Aki Vehtari, and Donald B Rubin. 2013. *Bayesian Data Analysis*. 3rd ed. Boca Raton, FL: CRC Press.
